# Identification of factors influencing tampering of codeine-containing medicines in England: a qualitative study

**DOI:** 10.1186/s12954-020-00408-w

**Published:** 2020-09-11

**Authors:** Andreas Kimergård, Stephen Parkin, Stacey Jennings, Eileen Brobbin, Paolo Deluca

**Affiliations:** 1 Addictions Department, King’s College London, 4 Windsor Walk, London, SE5 8BB UK; 2 Centre for Psychiatry, Queen Mary University of London, Old Anatomy Building, London, EC1M 6BQ UK

**Keywords:** Codeine, Medicine regulation, Tampering, Cold water extraction (CWE), Paracetamol Overdose

## Abstract

**Background:**

Tampering of psychoactive medicines presents challenges to regulation and public health. However, little is currently known about what influences the decisions to treat codeine-containing medicines (CCM) with cold water extraction (CWE) from the perspective of individuals employing these techniques. The article identifies factors influencing utilisation of CWE to separate codeine from compounded analgesics, such as paracetamol and ibuprofen, found in CCM.

**Methods:**

Purposive sampling of 27 participants residing in England who took part in a qualitative interview. Of these, 14 individuals (11 males and 3 females) reported tampering of psychoactive medicines, and the relevant transcripts were included in the analyses for the study. Participants were recruited from one addiction treatment service and from an online survey. The mean age of the participants was 31.5 years (range = 18–42 years). Qualitative data analysis followed the processes of iterative categorization (IC). The codes ‘harm reduction’, ‘information sources’ and ‘changes on the drug markets’ were grouped and summarised. The coding of the data was done in a Microsoft® Word document.

**Results:**

Two groups of participants were identified in the data analysis: (i) individuals who used CCM (*n* = 5), and (ii) individuals who used CCM and heroin (*n* = 9). Participants in both groups used CWE due to concerns of paracetamol overdose from the use of excessive dosages of CCM. For both of them, information obtained from the internet encouraged the use of CWE. Participants using CCM described how the many steps involved in conducting CWE, including sourcing codeine boxes from pharmacies (over the counter), presented a barrier against using CWE. Participants using CCM and heroin explained how reduced availability in the local heroin supply influenced utilisation of CWE techniques to maintain their use of opioids and avoid withdrawal. Withdrawal symptoms and cravings outweighed the concerns about the quality of the extracted codeine mixtures in this participant group, especially the ability of CWE to remove paracetamol and tablet fillers.

**Conclusions:**

Utilisation of CWE of codeine was influenced by several factors including drug market supply, the availability of detailed information on the internet about CWE and restrictions on codeine sourcing in pharmacies. Risks identified with CWE include consumption of unknown doses of paracetamol if the CWE techniques are not used correctly. Attempts at extracting codeine from CCM should be considered in risk assessments of opioid medicines.

## Introduction

Tampering of psychoactive medicines represents a challenge for the pharmaceutical industry, medicine regulators, community pharmacies and prescribers to prevent opioid-related harms such as overdose and dependence. Medicine tampering involves a range of procedures to manipulate different types of psychoactive medicines in order to enhance their psychoactive effects [1, 2]. Reports of harm following intravenous injection of crushed oxycodone and morphine tablets highlight the risk of pulmonary granulomatosis from deposition of talc and other fillers used in the manufacturing of medicines [3, 4]. Smoking of fentanyl patches (either whole patches or smaller pieces) have been reported amongst people seeking addiction treatment, setting new demands for services to negotiate severe withdrawal symptoms amongst their clients [5]. Studies have also shown that tablets are crushed and snorted [6], including tampering-resistant formulations which were released onto the market to make it harder to crush, snort and inject psychoactive medicines [7].

In England (and in many other countries) [8], codeine combined with a non-opioid analgesic (such as paracetamol or ibuprofen) are used to treat mild to moderate types of pain [9]. Codeine-containing medicines (CCM) containing small amounts of codeine present at the low end of the spectrum of analgesics that begins with non-opioids (such as paracetamol and ibuprofen), progressing to codeine combined with non-opioid analgesics sold over the counter (OTC) from licensed pharmacies, then to prescribed codeine of 15 mg and more (either alone or combined with paracetamol), and onto stronger psychoactive medicines such as tramadol, oxycodone and fentanyl for more severe kinds of pain. Some of the most commonly sourced OTC CCM contains 8 mg codeine and 500 mg paracetamol per tablet. Another OTC CCM on the market contains 12.8 mg codeine and 200 mg ibuprofen per tablet. Prescription-only CCM contain 15 mg, 30 mg, or 60 mg codeine and 500 mg paracetamol per tablet. According to the British National Formulary (BNF), the maximum daily dose of codeine is 240 mg [10]. For paracetamol and ibuprofen, the daily limits are 4000 mg paracetamol [11] and 2400 mg ibuprofen [12]. Exceeding those limits comes with a risk of harm such as hepatotoxicity [13].

In recent years, procedures for tampering with CCM, known as ‘cold water extraction’ (CWE), have emerged on the internet [2, 13, 14]. CWE usually involves crushing and dissolving tablets in water and then using home utensils such as coffee filters in order to separate the codeine from the accompanying paracetamol or ibuprofen (which both have potential for toxicity in doses exceeding the daily recommendations [15–17]). This can be done at home following relatively simple steps and enables people to ingest large quantities of codeine without the risk of paracetamol or ibuprofen overdose [14]. Reports of CWE from the UK date back to at least the mid-1980s where it was reported amongst people who used illicit opioids (‘street heroin’) [18]. Since then, an internet study which analysed reports posted on a public internet forum has described the use of CWE amongst its members [19]. Studies using recipes found in the grey literature and online have been published showing varying outcomes of treating CCM with CWE in regard to the amounts of medicines that are left in the extracted solutions [14, 20]. One study found that the extracted mixtures obtained from four tablets (suppositories) contained a mean of 94% codeine but they also contained more than 50% paracetamol (which was supposed to have been removed from CWE) [21]. We [14] reported significant variation in the amount of remaining non-opioid analgesics in codeine solutions extracted from three OTC CCM sold in the UK (two products) and Denmark (one product): the highest amounts ranged from 57 to 73% acetylsalicylic acid (CCM sold in Denmark); the lowest from 5 to 9.2% paracetamol with a recovery of 42–71% codeine from 19 tablets containing 8 mg codeine and 500 mg paracetamol per tablet (CCM sold in the UK); and 5.5–8.5% ibuprofen with 61–67% codeine from 12 tablets containing 12.8 mg codeine and 200 mg ibuprofen per tablet (another CCM sold in the UK) [14]. Extrapolation from experiment to CWE practices thus suggests that the CWE procedures enable consumption of approximately 1300 mg free-base codeine without exceeding the daily restriction of 2400 mg ibuprofen from CCM containing codeine and ibuprofen and approximately 500 mg free-base codeine from CCM containing codeine and paracetamol without exceeding the limit of 4000 mg paracetamol [14].^1^ As such, these procedures for CWE disrupt the regulatory system of psychoactive medicines by facilitating codeine use from CCM in much higher dosages than intended.

Reports of harms associated with CWE have been published elsewhere [22, 23], including reports of one fatality in which a pot and coffee filters were found near the fatality (toxicological analyses of blood and urine confirmed the presence of opioids) [24]. A case series of presentations at two London hospitals compiled data from six patients with self-reported ingestion of codeine or dihydrocodeine treated with CWE [13]. The number of tablets used for CWE of codeine ranged from 15 to 70 containing either 8 mg codeine or 12.8 mg codeine per tablet. None of them developed paracetamol toxicity. To date, however, there is lack of qualitative research to provide insights on the motives for and experiences of conducting CWE.

As one of the major consumers globally [25], codeine is widely available for use in the UK (sold OTC, prescribed, and dispensed as take-home boxes from Emergency Departments [26]), and therefore susceptible to tampering in the form of CWE amongst people who wish to consume excessive doses of codeine. As the literature suggests, people who consume codeine treated with CWE are at risk from exposure to unknown quantities of both codeine and accompanying analgesics (such as paracetamol, ibuprofen, and acetylsalicylic acid) [13, 14]. However, a better understanding of the experiences of CWE, and how CWE produces and reduces risk would contribute to evidence for risk assessments of opioid medicines and for the design of appropriate and effective public health responses.

During the period 2015 and 2016, we conducted a qualitative semi-structured interview study to gain further understanding of the factors which influence the development of codeine dependence in order to facilitate the design of optimal methods of prevention, intervention and treatment of codeine dependence [27]. This article reports findings from the same study, presenting an analysis of what influenced decisions to utilise CWE techniques according to the perspective of those who have attempted this procedure. This is the first known qualitative study with a primary focus on CWE. Tampering of CCM enables use of higher doses of codeine than intended from a regulatory perspective, facilitates use of opioids for other reasons than pain treatment and presents risks in form of paracetamol overdose. Understanding the decisions to conduct CWE can help inform harm reduction strategies.

## Methods

Ethical approval for the qualitative study was obtained from King’s College London, Psychiatry, Nursing & Midwifery Research Ethics Subcommittee and the NHS REC London–London Bridge.

### Recruitment and sampling

Participants for the study (*n* = 27) were recruited from an online survey (*n* = 17) launched to investigate the use, misuse, and dependence of CCM in the UK [28] and amongst addiction treatment service users from a residential rehabilitation service in England (*n* = 10). The participants recruited from the online survey participated voluntarily in the qualitative study by leaving their name and contact information in the online survey if they wished to be contacted by the qualitative researcher (AK). A leaflet was provided to people in the addiction treatment service informing them of the opportunity to take part in the study. All residents had the opportunity to discuss the study with the qualitative researcher (AK) in the service.

Eligible participants comprised people aged 18 years or over, who had taken any type of CCM in the last 12 months and who had ‘misused’ CCM according to the following definition: use of psychoactive medicines (in this case CCM) other than as directed or as indicated, whether wilful or unintentional, and whether it resulted in harm or not [29]. The definition includes ‘misuse’ of CCM amongst pain patients who develop codeine dependence as well as people taking excessive codeine doses recreationally. Both groups have been found to make use of CWE procedures [13, 19, 30]. Patterns of use to establish eligibility were examined for each potential participant according to their responses on the online survey or during part of the initial screening prior to the interviews. Of the 27 interviews, transcripts from all of those who reported tampering of psychoactive medicines were included in the analyses for the present article (*n* = 14). All participants resided in England.

### Data collection

The interviews were conducted during 2015–2016 by the qualitative researcher (AK). All participants in the study provided written consent to take part in the study. The same topic guide was used for participants recruited from the online survey and from the addiction treatment service. Participants were asked basic demographic information, initial use of CCM, patterns of CCM use, use of other drugs and psychoactive medicines, difficulties managing codeine use and their views on CCM regulation. For each of these topics, a series of questions were compiled. Table 1 includes a list of the specific questions which generated interview data about the tampering of CCM. The interviews lasted between 35 and 95 min and took place in the residential rehabilitation service, over the phone, and in public spaces convenient to individual participants (cafes, libraries, train stations). All participants received a £20 gift voucher as reciprocal payment for providing time and data to the study^2^.

**Table 1:** Interview questions generating interview data about tampering of CCM

Questions	Prompts
When did you first start taking codeine?	What prompted you to start? What products did you use? Was this prescribed or not? How did it make you feel? What were the effects? What were you thinking/doing?
Can you describe your pattern of codeine use over time?	How has your use of codeine changed (increased/ decreased; same/different products?) How do you explain the change of use?
Could you tell me about your current use of codeine?	What codeine products do you take and how much? When - time of day? Where? With whom?
What are your awareness of potential harm?	
Did you ever source information on codeine on the internet?	From where? Why/why not?

### Data management and analyses

The interviews were recorded digitally and transcribed verbatim. Primary analysis involved deductive coding (analysis based on theories or previous knowledge, or upon the topic guide) [32] guided by questions from the topic guide. These codes were supplemented by inductive codes (analysis based on what emerges from within the dataset itself and what individual participants say) [32] derived from topics within the data. This systematic coding of the data line-by-line was done in a Microsoft® Word document. Because the file containing the verbatim data extracts was relatively short, we opted to use Microsoft® Word instead of a qualitative software package such as NVivo or MAXQDA [33]. Secondary analysis followed the processes attached to Iterative Categorization (IC) [33]. IC is suitable for use with deductive and inductive codes and developed within the research field of substance addiction. After coding all 14 transcripts, the codes ‘harm reduction’, ‘information sources’ and ‘changes on the drug markets’ were exported into three separate Microsoft® Word documents and read, grouped and summarised as data codes. The data were compared according to the experiences of (i) participants using CCM versus (ii) participants using CCM and heroin. Tertiary analysis included an inductive approach which sought to identify patterns, associations and explanations within existing literature, theories and policies in order to discuss practical strategies and interventions aimed at reducing the harms associated with CWE. The findings below present a summary of this tertiary analysis.

## Results

From data analysis, two distinct groups of participants emerged: those who used CCM versus those who used CCM and heroin (Table 2). The first group typically started out by using CCM to treat different types of pain. However, over time many started using higher and higher doses and some became dependent (as a result of different environmental factors such as unsupervised and long-term codeine prescribing) [27]. The second group predominantly used heroin while CCM was used intermittently in periods of reduced availability in the heroin supply. Amongst those using CCM and heroin, the length of time using CCM was longer compared against those using CCM (9.7 years compared to 4.2 years) and more of them were male (88.9% versus 60.0%) (Table 2). Despite the difference between the two groups in terms of their drug using patterns, the procedures for CWE were described in largely the same way. However, while most of the participants reported using coffee filters, P7 below used a sock for improvised filtration:

I diluted them [CCM tablets] with some warm water. I swirled them around and then I got a sock over a cup, and slowly poured it in. I saw the white powder on top, and then it went into the cup. I took the sock off, threw the white powder away, poured in the orange juice, well tried it first, and then put the orange juice in and drank that.

P7, age 38, CCM and heroin use

**Table 2:** Participant characteristics

	Participants using CCM *n* = 5 (%)	Participants using CCM and heroin *n* = 9 (%)	All participants *n* = 14 (%)
Gender			
Male	3 (60.0)	8 (88.9)	11 (78.6)
Female	2 (40.0)	1 (11.1)	3 (21.4)
Mean age in years (range)	29.4 (18–42)	32.7 (26–38)	31.5 (18–42)
Mean length of time between initial codeine use and last time used in years (range)^a^	4.2 (1.5–9)	9.7 (1–16)	7.7 (1–16)
CWE, route of administration			
Drinking the solution	2 (40.0)	4 (44.4)	6 (42.9)
Injecting the solution	0 (0.0)	3 (33.3)	3 (21.4)
Snorting crushed codeine tablets	0 (0.0)	2 (22.2)	2 (14.3)
CWE, reasons for use			
To make codeine work quicker	1 (20.0)	0 (0.0)	1 (7.1)
To feel euphoric	2 (40.0)	0 (0.0)	2 (14.3)
To prevent withdrawal symptoms	1 (20.0)	2 (22.2)	3 (21.4)

^a^For participants reporting current use the length of time was calculated as the difference between initial use and the date the interview was conducted

Having provided this general account of the steps involved in CWE, below we report the experiences of doing CWE to illustrate the key points. Table 3 provides a summary of the similarities and differences between the experiences of each group of participants (CCM versus CCM and heroin).

**Table 3:** Decisions to utilise CWE across groups of participants (CCM alone versus CCM and heroin)

Participants using CCM	Participants using CCM and heroin	All participants
		CWE was used to eliminate non-opioid analgesics (paracetamol and ibuprofen) to reduce the risk of paracetamol overdose.
The many steps involved (including sourcing OTC CCM from multiple pharmacies) made CWE less attractive.		
	Cravings outweighed concerns about the ineffectiveness of CWE and triggered utilisation.	
		Online information influenced use of CWE (facilitated tampering of psychoactive medicines but also reduced the risk of harm associated with paracetamol overdose).
	Concerns of injecting tablet fillers in solutions containing crushed tablets facilitated use of CWE.	
	When stronger codeine (prescription-only CCM) were not accessible, participants treated OTC codeine-containing tablets with CWE.	
	Reduced supply of heroin would lead participants to source other types of opioids including utilisation of CWE.	

### Harm reduction (CCM and heroin)

In both groups of participants, factors that influenced CWE related to concerns of paracetamol overdose and stemmed from the risks of ingesting excessive dosages of CCM containing codeine and paracetamol (or codeine combined with ibuprofen). Many of the participants who used CCM and heroin reported consuming more CCM tablets than recommended per day because they wanted to prevent withdrawal symptoms from their use of heroin. For them, CWE was used to reduce risks of harms to the liver:

I would be doing say definitely more tablets than eight grams of paracetamol, which is toxic.^3^ So, if I had ‘necked’ them [swallowed the tablets whole] I would have been looking at serious liver damage.

P25, age 30, CCM and heroin use

Other participants commented that cravings for opioids sometimes outweighed their concern of paracetamol overdose when they considered how effective CWE was in terms of removing the paracetamol. Underpinning such experiences, some participants cautioned that white powder and other residue that was left in the extracted solutions compromised the purity of the codeine extract. However, these visual assessments of its quality were not always enough to deter them from ingesting the solution.

I actually thought to myself, am I doing this right? I saw that white powder and I thought you know there has got to be some paracetamol left in there. But I didn’t care.

P7, age 38, CCM and heroin use

### Harm reduction (CCM)

The perceived ineffectiveness of CWE procedures did not always prevent consumption of the extracted codeine amongst the participants using CCM and heroin. However, amongst the participants who only used CCM, there were comments suggesting that all the steps required for CWE such as from collecting codeine boxes from multiple pharmacies to crushing them up, dissolving them in water and completing the filtration presented as a barrier to using CWE^4^^,^^5^. Considering that pharmacies are allowed to only dispense one box per day per customer, scouring enough boxes would require visits to more than one pharmacy on the same day [9]. This emerged as one of the key differences between the two groups, and it was especially common amongst those dependent on codeine who needed to use daily.

It was more the time, that you would have to do it properly and I didn’t have the patience. You know once you want a ‘fix’ you don’t have time to faff around.

P8, age 27, CCM use

Given these circumstances, some of the participants with probable codeine dependence seemed to have secured ways of collecting CCM from a medical prescription and from their friends and associates (diversion from legitimate use). These ways of gaining access to prescription-only CCM would enable consumption of sufficiently high codeine doses to avoid withdrawal without the risk of paracetamol overdose because of the more favourable ratio between codeine and paracetamol in prescription-only CCM compared to in OTC CCM.

### Information sources (CCM and heroin)

Participants understood that the risks of using high doses of paracetamol from CCM could be reduced by following the instructions for CWE which many found online. They explained further how they believed that CWE could also be used to remove tablet fillers before injecting the extracted codeine solutions. Such considerations were only found in the group of participants using both CCM and heroin as they were the only participants reporting injecting the extracted codeine solutions. Participants’ explanations also illustrated that the availability of detailed information about CWE methods facilitated the tampering of psychoactive medicines.

Tablets aren’t meant to be injected. They’re full of fillers for that reason. But I just thought there has to be a way around it. So yeah, I looked online and there was the whole CWE process. I suppose the internet helped me do that in a safer manner than perhaps I would have done. But then I have to ask would I have actually done it if I hadn’t found a safe method to do it? And I’m not sure about that.

P16, age 30, CCM and heroin use

### Information sources (CCM)

Participants considered the available sources of information about CWE. Word of mouth communication and information sourced online encouraged participants in both groups to utilise CWE, especially if they said they had learned about CWE on internet forums dedicated to drug use and related practices:

If I am doing something new or going to a new restaurant, I like to Google things…The same with medication. If you go on the drugs forums… For codeine, there’s the threads at the top, the sticky posts at the top, they’re all about CWE and warnings about paracetamol.

P12, age 18, CCM use

For some of the participants, seeking information online had close ties to how they would source their CCM for CWE. They told how they had discovered new products on the market including CCM sold as veterinary medicines. This presents an example of how online information and supply offer new venues of codeine sourcing which may not previously have been discovered nor accessible. As described in the previous theme, participants would compare the amounts of codeine and paracetamol in the different types of CCM that were available to them with an eye toward the products containing the most favourable ratio. Sourcing products with high amounts of codeine and low amounts of paracetamol for CWE played an important role in harm reduction:

Online you can order codeine, 500 tablets, 9 mg codeine and 400 mg paracetamol, which is like a better ratio than the over the counter ones,^6^ but it’s not designed for humans, it’s designed for dogs.

P12, age 18, CCM use

### Changes in drug markets (CCM and heroin)

Some of the participants outlined that the decision to consider CWE procedures was influenced by access to different types of CCM that contained a more or less favourable share of codeine versus paracetamol. For example, prescription-only CCM were available from diversion from legitimate uses and sources and supplied on the illicit drug market. Access to these formulations would eliminate the need to treat OTC codeine with CWE amongst those using CCM and heroin. By taking prescription-only codeine participants could consume sufficiently high doses of codeine without exceeding the maximum recommendations for paracetamol. In contrast, P25 below explained that only having access to OTC CCM would mean having to resort to CWE to consume sufficient amounts of codeine to avoid withdrawal:

My friend at the time he couldn’t get the stronger tablets anymore [30 mg codeine and 500 mg paracetamol per tablet]. It was either extraction [from OTC CCM of 8 mg codeine and 500 mg paracetamol per tablet] or don’t get codeine at all.

P25, age 30, CCM and heroin use

Further, participants in this group also reflected on the links between their use of heroin and the use of codeine obtained from CWE. They noted that doing CWE served multiple purposes relating to both injecting practices and consuming opioids during reduced availability in the local heroin supply. This is also illustrative of participants who sometimes injected the extracted codeine solutions:

As well as the heroin I was missing, I was also missing the ritual of IV use [injecting in the vein]. So, it was to plug the gap that the drought was leaving and also scratch the itch that I was having for not having that whole ritual of injecting. It was something that taking codeine orally just didn’t do.

P16, age 30, CCM and heroin use

## Discussion

This qualitative study explored what influenced decisions to utilise CWE to gain further knowledge about the tampering of psychoactive medicines in England. Such findings are useful for comprehensive risk assessments of opioid medicines [35].

We describe and compare the experiences of two groups of participants: (i) those who only used CCM and (ii) those who used CCM and heroin. Our analyses highlight that concerns of paracetamol overdose from CCM informed decisions to treat codeine tablets with CWE in both groups of participants. The prospect of removing paracetamol from CCM reflects prior research on factors which encouraged dissemination of CWE techniques on online drug forums [19]. Drug market behaviours, such as reduced availability in the local supply of heroin, informed participants’ utilisation of CWE amongst those using CCM and heroin. In circumstances where prescription-only codeine (which contain a higher ratio of codeine versus paracetamol than OTC CCM) were temporarily unavailable from diversion, participants using CCM and heroin resorted to CWE of OTC codeine to continue their use of high codeine dosages without the risk of paracetamol overdose. The only barrier acting against this form of tampering of psychoactive medicines found in the study was the amount of time required to collect enough boxes of OTC codeine and the amount of time required to treat codeine with CWE. This was evident amongst participants who only used CCM. Information about CWE online was accessed by all the participants and assisted uptake. From a harm reduction perspective, the possibility of removing paracetamol from CCM protected participants against the harms associated with paracetamol overdose (such as hepatotoxicity). More so, this was the case amongst the participants who had no alternative sources of opioids (other than OTC CCM) and were not ready to cease their use. However, the variability of the amounts of paracetamol and ibuprofen left in extracted mixtures (typically of an unknown quantity to people practicing CWE) constitute a risk [14, 20, 21]. As such, the ease at which codeine can be obtained, their potential for tampering and the existence of recipes for CWE represent factors exposing people who are treating CCM with CWE to harm.

Findings pertaining to the awareness of the harms associated with the non-opioid analgesics found in CCM highlight concerns of paracetamol overdose from the use of excessive doses and resonates with prior qualitative research on risk awareness amongst people with probable codeine dependence [30, 36]. The influence of risk awareness on drug taking behaviours, included information gathering and taking precautions such as treating CCM with CWE. Changes in behaviour triggered by reports of harms and risks and subsequent dissemination of harm reduction drug using practices online and by word of mouth amongst people who use CCM resonate with reports of information-seeking and associated behaviour change amongst other populations of people who are using opioids. There is evidence to support that people who use drugs will access information from multiple sources including television, from other people who use drugs, friends, relatives, online and print media to protect themselves from harm occurring [37, 38].

Some of our findings identify issues which have been less well-described in the scientific literature. For example, in this study, participants’ decisions to conduct CWE were influenced by the supply of illicit opioids on the local drug markets. Previous research has shown that fluctuation in the heroin supply may trigger displacement activities such as seeking treatment and quitting, or using alcohol and benzodiazepines and diverted opioids such as methadone [39–41]. Our findings suggest displacement to codeine extracted from OTC CCM during periods of reduced availability in the heroin supply, or when prescription-only CCM were unavailable, otherwise undetected in empirical research [30, 36]. These findings may reflect law enforcement activity targeting markets for heroin and stronger pharmaceutical opioids. They may also reflect changes in the commissioning and provision of structured addiction treatment resulting in increased unmet treatment needs leading to continued opioid use with sourcing of multiple types of opioids to avoid withdrawal and cravings as the result. As such, the findings provide an empirical example of the ‘risk environments’ thesis by Rhodes [42], involving the impact of macro, meso and micro environments on the production of drug-related harms.

For all participants, failed attempts at CWE if the techniques are not used correctly means that high amounts of paracetamol in the extracted solutions could remain. Likewise, variation in employing the CWE procedures, for example by switching between different brands of coffee filters for each attempt at extraction or cooling the solutions to different temperatures each time, will likely yield varying results over consecutive attempts and lead to inconsistencies between the amounts of codeine and paracetamol (or ibuprofen) left in the extracted solutions [14, 21, 24]. This represents a risk of harm from paracetamol overdose such as hepatoxicity. Research also suggests that some types of CCM treated with CWE yields higher amounts of codeine and lower amounts of paracetamol than other CCM [14, 21]. This may not be known to people practising CWE.

For the participants using CCM and heroin, it is important to note that no studies, as far as we are aware, have investigated the content of extracted solutions from CWE in regard to left over tablet fillers (such as talc or starch used in the manufacturing of pharmaceutical tablets). Tablet fillers can cause serious harm when injected intravenously, including infections at the injection site and pulmonary emboli [4, 43]. This poses a risk specifically to those who inject the extracted codeine.

Diversion of medicines fuelled the availability of opioids on the illicit markets and played a role in decisions to treat codeine with CWE. Prescribers should familiarise themselves with the types of psychoactive medicines that appeal to tampering procedures and report tampering to the UK Medicines & Healthcare Products Regulatory Agency which could serve to limit diversion from ‘doctor shopping’ [44, 45]. Importantly to the public health efforts initiated to curb the number of people experiencing dependence and withdrawal from psychoactive medicines [46], however, is that any changes in formulation of CCM or other regulatory intervention dramatically and abruptly affecting the present availability of CCM should take into consideration potential displacement to other opioids. Furthermore, amongst people who are opioid dependent regulatory steps affecting the availability of CCM should be accompanied by offers of interventions and treatment of opioid dependence [47].

### Strengths and limitations

A limitation of this study is the small sample size of 14 participants. However, findings from qualitative research is not supposed to be empirically generalisable but instead have transferability to other contexts by relating patterns and themes to a known body of knowledge [33]. Data saturation at which no new data about a particular issue (in this case CWE) is raised in successive interviews [48] was not achieved due to the small sample size. The sample included in the analysis for the current article were predominantly male (78.6%) whereas the full sample of all participants recruited to the study (*n* = 27) had a gender distribution of 14 females (51.9%) and 13 males (49.1%) [27]. Furthermore, out of the nine participants who reported CCM and heroin use, 88.9% were male shifting the gender distribution even further. Given that there is a lack of sufficient data in the topic of tampering of psychoactive medicines in England, we cannot say how the gender distribution in our sample of 14 participants reflects the gender distribution in larger cohorts tampering with psychoactive medicines in England. However, indicators of high-risk drug taking that may possibly involve tampering of psychoactive medicines such as intravenous injections of crushed and dissolved tablets reveal that a majority of addiction service treatment entrants for opioids are male (2017/2018) [49]; a majority of drug-related deaths occurred amongst male in 2018 [50]. In a case series reporting patients presenting to Emergency Departments in England with reported consumption of codeine and dihydrocodeine extracted from CWE all six patients were male [13]. As such, the sample included in our analyses may indeed reflect the characteristics of subpopulations engaging in this form of tampering of psychoactive medicines. Another limitation is that findings from the study cannot be generalised to all regions of England. For example, variability in the use and local supply of heroin may impact the use of CWE differently across regions.

## Conclusion

The article provides new information about the tampering of psychoactive medicines in England in a field where data about CWE of codeine from CCM are scarce and calls attention to tampering techniques for risk assessments of opioid medicines. Many factors influenced decisions to treat CCM with CWE. For both groups of participants, concerns of paracetamol overdose from excessive CCM consumption as well as the availability of recipes for CWE on the internet influenced decisions to do CWE to reduce harm such as hepatoxicity. In those who only used CCM, the number of steps involved in extracting codeine required too much time and effort to make it worth the while. Amongst those using CCM and heroin, CWE played a role in maintaining opioid use to avoid withdrawal during times of reduced availability of heroin. Overall, CWE methods seemed improvised and a challenge to harm reduction and yet the participants appeared knowledgeable about CWE and how to avoid the physical risk and harms of paracetamol overdose. Globally, many implemented risk minimisation strategies have focussed on stronger opioids than CCM (such as fentanyl, tramadol and oxycodone) [51]. Yet, our study suggests that CCM pose a challenge in the availability of opioids by contributing to dependence, tampering of psychoactive medicines and problematic drug use in England involving both pharmaceutical opioids and illicit opioids (heroin). Risk minimisation strategies need to take into account the possibility of CWE from CCM resulting in unknown doses of paracetamol if the techniques are not used correctly.

## Data Availability

The dataset generated and analysed during the current study are not publicly available due to the respect of individual privacy.

## Abbreviations

OTC: Over the counter
CCM: Codeine-containing medicines
CWE: Cold water extraction
BNF: British National Formulary
IC: Iterative categorization

## Footnotes

1 In layman’s terms, this means that CWE enables individuals to drink about five times the daily dose of codeine in one cup of extracted codeine solution.

2 Participants received vouchers, as cash can easily be spent on CCM, drugs or alcohol. Furthermore, we opted to use vouchers to avoid having to carry large amounts of cash when conducting multiple interviews on the day or in the same area. However, for a discussion of the possible benefits of using cash as reciprocal payment, see Neale et al. [31].

3 8000 mg paracetamol is twice the recommended daily dose according to the BNF.

4 To provide context for interpretation, participants would require two boxes of OTC CCM (or about 60 tablets containing 8 mg codeine and 500 mg paracetamol) to produce approximately 500 mg free-base codeine (maximum achievable dose from CWE without exceeding the upper limit of paracetamol in the extracted solution). At a price of approximately £1 for a box of OTC CCM (32 tablets containing 8 mg codeine and 500 mg paracetamol), the cost of producing approximately 500 mg free-base codeine is less than £2. In comparison, the price of 1 bag of heroin is estimated to £10 in the UK (although the weight may be reduced or unknown to sellers and buyers) [34]).

5 To produce 1300 mg free-base codeine from CWE (maximum achievable dose without exceeding the upper limit of ibuprofen), participants would require three boxes of OTC CCM (or around 96 tablets containing 12.8 mg codeine and 200 mg ibuprofen).

6 A comparable OTC CCM intended for human consumption contains 8 mg codeine and 500 mg paracetamol.

## References

[1] Kimergård A, Breindahl T, Hindersson P, Deluca P (2016). Tampering of opioid analgesics: a serious challenge for public health?. Addiction..

[2] Cone EJ (2006). Ephemeral profiles of prescription drug and formulation tampering: evolving pseudoscience on the Internet. Drug Alcohol Depend.

[3] Anderson RJ, Corbett B, Ly BT (2016). A case of acute pericarditis following intravenous injection of crushed morphine tablets. J Psychoactive Drugs.

[4] Naik P, Cashin L, Huitron S (2016). A case of pulmonary foreign body granulomatosis secondary to intravenous injection of acetaminophen/oxycodone. Mil Med.

[5] Kimergård A, Dunne J, Bøgen A, Hindersson P, Breindahl T (2018). Characteristics of opioid-maintained clients smoking fentanyl patches: The importance of confirmatory drug analysis illustrated by a case series and mini-review. Drug Test Anal.

[6] Vorce SP, Levine B, McDonough PC, Past MR (2010). An overdose death involving the insufflation of extended-release oxymorphone tablets. J Anal Toxicol.

[7] Leece P, Orkin AM, Kahan M (2015). Tamper-resistant drugs cannot solve the opioid crisis. Can Med Assoc J.

[8] Foley M, Kelly P, Deluca P, Kimergård A (2018). Advertising of over-the-counter codeine-containing medicines in the EU: differences in the regulation of advertising between member states. Pharm Med.

[9] Foley M, Carney T, Rich E, Parry C, Van Hout M-C, Deluca P (2016). Medical professionals’ perspectives on prescribed and over-the-counter medicines containing codeine: a cross-sectional study. BMJ Open.

[10] National Institute for Health and Care Excellence. Codeine phosphate [Internet]. National Institute for Health and Care Excellence; 2020 [cited 2020 Mar 5]. Available from: https://bnf.nice.org.uk/drug/codeine-phosphate.html.

[11] National Institute for Health and Care Excellence. Paracetamol [Internet]. National Institute for Health and Care Excellence; 2020 [cited 2020 Mar 9]. Available from: https://bnf.nice.org.uk/drug/paracetamol.html.

[12] National Institute for Health and Care Excellence. Ibuprofen [Internet]. National Institute for Health and Care Excellence; 2020 [cited 2020 Mar 9]. Available from: https://bnf.nice.org.uk/drug/ibuprofen.html.

[13] Harnett JT, Dines AM, Wood DM, Archer JRH, Dargan PI (2020). Cold water extraction of codeine/paracetamol combination products: a case series and literature review. Clin Toxicol.

[14] Kimergård A, Deluca P, Hindersson P, Breindahl T (2016). How resistant to tampering are codeine containing analgesics on the market? Assessing the Potential for Opioid Extraction. Pain Ther.

[15] Frei MY, Nielsen S, Dobbin MDH, Tobin CL (2010). Serious morbidity associated with misuse of over-the-counter codeine–ibuprofen analgesics: a series of 27 cases. Med J Aust.

[16] Cock V, Edmonds C, Cock C (2018). Complications related to chronic supratherapeutic use of codeine containing compound analgesics in a cohort of patients presenting for codeine withdrawal: Codeine analgesic complications. Drug Alcohol Rev.

[17] Robinson GM, Robinson S, McCarthy P, Cameron C. Misuse of over-the-counter codeine-containing analgesics: dependence and other adverse effects. 2010;123(1317):6. 20657632

[18] Sakol MS, Stark CR (1989). Codeine abuse. Lancet.

[19] Van Hout MC (2015). Nod and wave: An Internet study of the codeine intoxication phenomenon. Int J Drug Policy.

[20] Fleming GF, McElnay JC, Hughes CM (2003). The Separation of Codeine from Nonprescription Combination Analgesic Products. Subst Use Misuse.

[21] Pascali JP, Fais P, Vaiano F, Pigaiani N, D’Errico S, Furlanetto S (2018). Internet pseudoscience: Testing opioid containing formulations with tampering potential. J Pharm Biomed Anal.

[22] Donuk T, Altıntoprak AE, Tekin H (2016). Possible causal link between idiopathic intracranial hypertension and the misuse of codeine-based products. J Child Adolesc Psychopharmacol.

[23] Stewart C, McGlen S (2019). Pharmacy staff should be aware of drug misusers’ methods of extracting opioids from OTC products. Clin Pharm.

[24] Fais P, Pigaiani N, Cecchetto G, Montisci M, Gottardo R, Viel G (2017). “tampering to death”: a fatal codeine intoxication due to a homemade purification of a medical formulation. J Forensic Sci.

[25] International Narcotics Control Board (2018). Narcotic Drugs: Estimated World Requirements for 2019.

[26] Raman R, Sinclair A, Kimergård A (2019). The prescription opioid epidemic: the role of Emergency Departments in the UK compared to the USA. Gateshead United Kingdom.

[27] Kinnaird E, Kimergård A, Jennings S, Drummond C, Deluca P (2019). From pain treatment to opioid dependence: a qualitative study of the environmental influence on codeine use in UK adults. BMJ Open.

[28] Kimergård A, Foley M, Davey Z, Dunne J, Drummond C, Deluca P (2017). Codeine use, dependence and help-seeking behaviour in the UK and Ireland: an online cross-sectional survey. QJM Int J Med.

[29] EMCDDA (2016). Strategies to prevent diversion of opioid substitution treatment medications.

[30] Van Hout MC, Horan A, Santlal K, Rich E, Bergin M (2018). ‘Codeine is my companion’: misuse and dependence on codeine containing medicines in Ireland. Ir J Psychol Med.

[31] Neale J, Black L, Getty M, Hogan C, Lennon P, Lora C (2017). Paying participants in addiction research: Is cash king?. J Subst Use.

[32] Becker S, Bryman A, Ferguson H (2012). Understanding research for social policy and social work: themes, methods and approaches.

[33] Neale J (2016). Iterative categorization (IC): a systematic technique for analysing qualitative data: Systematic technique for analysing qualitative data. Addiction..

[34] Shapiro H, Daly M (2017). Highways and buyways: A snapshot of UK drug scenes 2016.

[35] MHRA. Opioid Expert Working Group meets at MHRA [Internet]. MHRA; 2020 [cited 2020 Mar 3]. Available from: https://www.gov.uk/government/news/opioid-expert-working-group-meets-at-mhra.

[36] Cooper RJ (2013). ‘I can’t be an addict. I am.’ Over-the-counter medicine abuse: a qualitative study. BMJ Open.

[37] Freeman RC, French JF (1995). What Is the Addicts’ Grapevine When There’s ‘Bad Dope’: An Investigation in New Jersey. Public Health Rep.

[38] Mars SG, Ondocsin J, Ciccarone D (2018). Sold as Heroin: Perceptions and Use of an Evolving Drug in Baltimore, MD. J Psychoactive Drugs.

[39] Ahmad M, Richardson A (2010). Impact of the reduction in heroin supply between 2010 and 2011.

[40] Fountain J, Strang J, Gossop M, Farrel M, Griffiths P (2000). Diversion of prescribed drugs by drug users in treatment: analysis of the UK market and new data from London. Addiction..

[41] Fraser A, George M (1988). Changing Trends in Drug Use: an initial follow-up of a local heroin using community. Addiction..

[42] Rhodes T (2009). Risk environments and drug harms: A social science for harm reduction approach. Int J Drug Policy.

[43] Roux P, Carrieri MP, Keijzer L, Dasgupta N (2011). Reducing harm from injecting pharmaceutical tablet or capsule material by injecting drug users: Microfilters for injecting drug users. Drug Alcohol Rev.

[44] Sansone RA, Sansone LA (2012). Doctor shopping: A phenomenon of many themes. Innov Clin Neurosci.

[45] Chenaf C, Kabore J-L, Delorme J, Pereira B, Mulliez A, Roche L (2016). Codeine shopping behavior in a retrospective cohort of chronic noncancer pain patients: incidence and risk factors. J Pain.

[46] Taylor S, Annand F, Burkinshaw P, Greaves F, Michael K, Knight J (2019). Dependence and withdrawal associated with some prescribed medicines: an evidence review.

[47] Ciccarone D (2019). The triple wave epidemic: supply and demand drivers of the US opioid overdose crisis. Int J Drug Policy.

[48] Saunders B, Sim J, Kingstone T, Baker S, Waterfield J, Bartlam B (2018). Saturation in qualitative research: exploring its conceptualization and operationalization. Qual Quant.

[49] Public Health England (2018). Adult substance misuse statistics from the National Drug Treatment Monitoring System (NDTMS).

[50] Office for National Statistics (2019). Deaths related to drug poisoning in England and Wales: 2018 registrations.

[51] Vosburg SK, Jones JD, Manubay JM, Ashworth JB, Shapiro DY, Comer SD (2013). A comparison among tapentadol tamper-resistant formulations (TRF) and OxyContin ® (non-TRF) in prescription opioid abusers: Assessment of tapentadol TRF. Addiction..

